# Displacement Estimation Error in Laser Scanning Monitoring of Retaining Structures Considering Roughness

**DOI:** 10.3390/s21217370

**Published:** 2021-11-05

**Authors:** Hyungjoon Seo, Yang Zhao, Cheng Chen

**Affiliations:** 1 Department of Civil Engineering and Industrial Design, University of Liverpool, Liverpool L69 3BX, UK; 2 Department of Civil Engineering, Xi’an Jiaotong Liverpool University, Suzhou 215000, China; yang.zhao@xjtlu.edu.cn (Y.Z.); cheng.chen19@student.xjtlu.edu.cn (C.C.)

**Keywords:** point cloud, laser scanning, retaining structure, C2C, C2M, M3C2

## Abstract

Point clouds were obtained after laser scanning of the concrete panel, SMW, and sheet pile which is most widely used in retaining structures. The surface condition of the point cloud affects the displacement calculation, and hence both local roughness and global curvature of each point cloud were analyzed using the different sizes of the kernel. The curvature of the three retaining structures was also analyzed by the azimuth angle. In this paper, artificial displacements are generated for the point clouds of 100%, 80%, 60%, 40%, and 20% of the retaining structures, and displacement and analysis errors were calculated using the C2C, C2M, and M3C2 methods. C2C method is affected by the resolution of the point cloud, and the C2M method underestimates the displacement by the location of the points in the curvature of the retaining structures. M3C2 method had the lowest error, and the optimized M3C2 parameters for analyzing the displacement were presented.

## 1. Introduction

Retaining structures have been used in various excavation sites to prevent the failure of soils. Precast concrete panels are widely used when reinforcing methods need to be applied after excavation is completed. The lateral movement of the ground can be suppressed by being used with reinforcement methods, such as anchors and soil nailing in concrete panels [1,2,3,4,5,6,7,8]. The resistance between soil and grouting can be generated during the movement of soil [9,10,11]. If the displacement of the ground has a great influence on the stability of the entire construction, a retaining structure can be installed before excavation. Soil mixing wall (SMW), which reinforces the ground using grouting, is constructed in the vertical direction before excavation and then minimizes the lateral displacement by excavation [12]. Sheet piles are one of the most widely used in retaining structures in recent years because the penetration of groundwater can be minimized at the site where sheet piles are installed [13,14]. Construction methods, such as anchors and soil nailing can be used with sheet piles to suppress displacement. Each retaining method is constructed for the purpose of minimizing displacement by excavation. Therefore, displacement has to be continuously monitored.

Various monitoring techniques have been applied to control the displacement in excavation sites. Monitoring techniques, such as inclinometers, extensometers and total stations have been used to measure vertical and horizontal displacements [15]. In order to determine the stability of retaining structures and reinforcing materials, strain gauges have been installed to measure the strain of each structure. However, the existing monitoring techniques can only monitor a specific point where the sensor is installed, and the installed sensors cannot be monitored for a long time due to groundwater and environmental influences. Recently, with the development of various SMART monitoring techniques, techniques that can measure permanent and wide sections from one dimension to three dimensions instead of specific points have been being developed [16]. Since the optical fiber sensor can be used permanently, studies to measure the strain of the infrastructure for the long term have been conducted. The distributed optical fiber sensor can continuously measure the strain of cables for more than 20 km, and hence it is recently applied as a SMART monitoring technique for various infrastructures [17,18,19]. In order to monitor displacement in wide sections at large-scale excavation construction sites, photogrammetry techniques are being introduced [20,21,22]. Research on applying thermal infrared cameras has been conducted to find local damage, such as cracks in structures [23,24]; 3D cameras have been also applied to evaluate the global movement of structures’ environmental area [25]. However, the accuracy of monitoring is not enough to detect the displacement of some infrastructure because the resolution of monitoring is determined by the distance between the structure and the 3D camera [26]. Recently, studies have been conducted to measure the deformation of structures using 3D laser scanning [27,28,29,30]. Since 3D laser scanning has higher resolution and accuracy than other imaging techniques, it can be applied to infrastructures where displacements in mm units occur, such as at excavation sites; 3D laser scanning can scan the entire structure within a short time, and hence the behavior of the entire structure can be evaluated using a technique, such as a displacement mapping [31].

Point clouds are obtained by laser scanning, since numerous points discontinuously represent a structure, the effect of resolution causes a major error in the monitoring of results. The Cloud to Could (C2C) comparison method, which directly compares points and points, is most affected by resolution. In order to minimize the effect of the resolution, techniques, such as Cloud to Mesh (C2M), which analyzes the distance after representing the point cloud as a mesh, have been developed [32]. Since the point cloud contains information of lots of points, the accuracy of monitoring can be improved by an analysis method. When laser scanning is applied as a monitoring technique for a specific structure, it is essential to define the range of errors that a specific analysis method has. Therefore, this paper attempts to estimate the analysis errors that can occur when various analysis methods are applied to the displacement estimation of retaining structures. The distance analysis error can also be greatly influenced by the surface roughness and curvature of retaining structures. In this paper, after collecting the point cloud of retaining structures, such as concrete panel, sheet pile, and SMW, the distance analysis errors were evaluated in three retaining structures. This paper presents the errors that can occur by each displacement analysis technique when laser scanning is used for retaining structures. Therefore, this paper not only increases the applicability of laser scanning to the displacement monitoring of retaining structures in the future but also the calculated error can be applied when monitoring the displacement of the retaining structure by laser scanning.

## 2. Laser Scanning and Analysis Methods in Different Types of Retaining Structure

**Figure 1:**
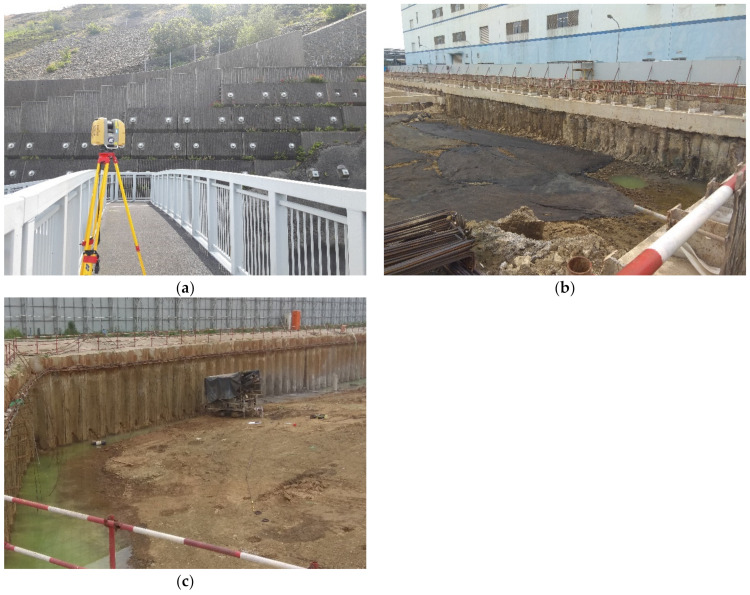
Laser scanning of different retaining structure types, (**a**) Concrete panel;(**b**) Soil mixing wall;(**c**) Sheet pile.

In this paper, laser scanning was performed to collect the point clouds of concrete panels, SMW, and sheet piles, which are widely used as retaining structures. The concrete panel was constructed with an anchor as shown in Figure 1a, and the surface of the concrete panel was rough and zigzag. SMW is a support material during excavation after completion of the construction before excavation. SMW has a rough surface and random curvature because the soil and grouting are mixed (see Figure 1b). Since the surface of the SMW is exposed after the excavation is completed, not only the SMW body but also the residual soil can remain on the excavated surface of the SMW. Sheet piles are also constructed before excavation so that the surface is exposed after excavation. However, the surface of the sheet piles is flat and the residual soil from the excavation is also less than that of the SMW (see Figure 1c).

Since the three retaining structures are scanned by a laser scanner in this paper they have different surface features, the displacement of the point cloud can be affected by surface conditions during the analysis. The laser scanner used in this paper is a Leica P40 and Topcon GLS-2000. The range accuracy of the Leica P40 is 1.2 mm + 10 ppm, and the maximum resolution is 0.8 mm at 10 m. The accuracy of the single point of Topcon GLS-2000 is 3.5 mm, and the maximum resolution is 3.1 mm at 10 m.

**Figure 2:**
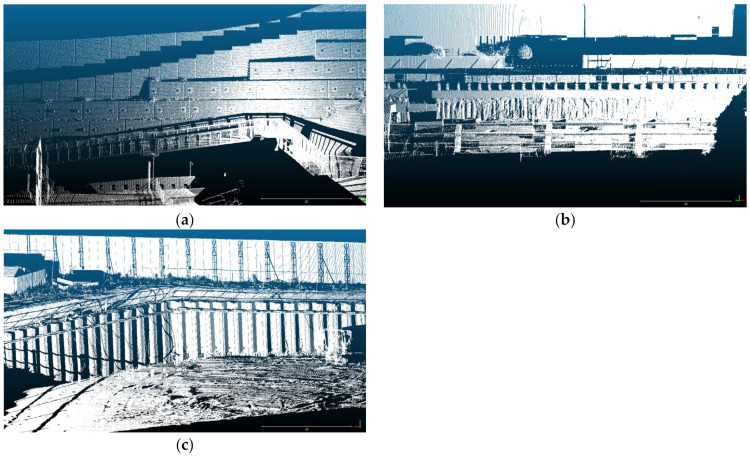
Point clouds of different retaining structure types, (**a**) Concrete panel (**b**) Soil mixing wall (**c**) Sheet pile.

Figure 2 shows the raw data of each retaining structure obtained by laser scanning. Figure 2a shows a point cloud scanned over the entire retaining structure composed of concrete panels. The retaining structure shown in the point cloud is 35 m wide and 10 m long. Since the concrete panel was manufactured as precast concrete at the factory, it has a regular shape but has a rough surface. The point cloud of the concrete panel includes the surface of the anchor face installed with the concrete panel. The surface of the SMW is exposed according to excavation. Therefore, the curvature and roughness of the surface are randomly distributed. SMW is constructed in a cylindrical shape after drilling in a vertical direction, but the shape is not uniform because the soil and grouting are mixed. The retaining structure of SMW shown in the point cloud is 30 m wide and 4 m long. The point cloud of the retaining structure composed of sheet piles is shown in Figure 2c. The sheet pile site is a large-scale retaining structure that excavates a section of more than 200 m, and the point cloud shown in Figure 2c represents some of them. Sheet piles not only have a regular shape but also have a flat surface despite being constructed before excavation.

## 3. Roughness Evaluation of Point Cloud in Retaining Structures

### 3.1. Pretreatment for Cloud Compares

**Figure 3:**
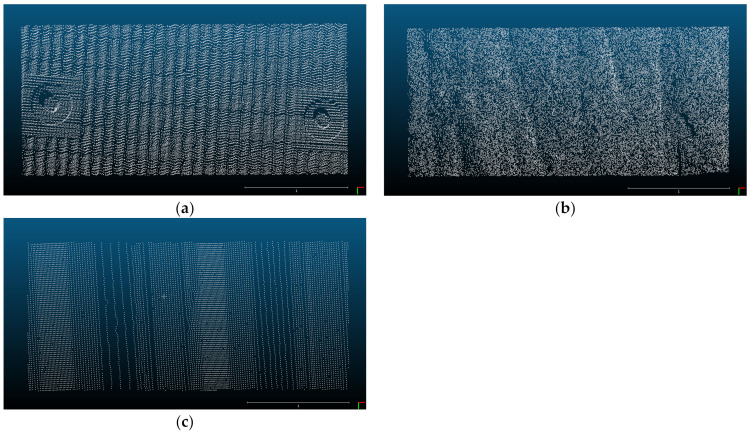
Equivalent cutting of point clouds, (**a**) Concrete panel, (**b**) Soil mixing wall, (**c**) Sheet pile.

The field conditions and laser scanning settings: In order to perform equivalent analysis of each point cloud, pre-treatment of the point clouds are required. The point clouds of each retaining structure are extracted in a size of 3.2 m in width and 1.45 m in length. Since the number of points in the extracted point clouds was all different, the number of points in the sheet pile and SMW were equally selected by random extraction according to the point cloud of the concrete panel which has the lowest number of points. Figure 3 shows the extracted point clouds of each retaining structure that all have the same point. Point clouds can be collected by scanning each of the retaining structures, and Figure 2 shows an example of the acquired point clouds. As shown in Figure 2a, the concrete panel is 2.1 m wide and 1.0 m long, and the shadowed area of the point cloud appears due to the curvature and roughness of the surface. This paper investigated the influence of the point cloud analysis due to such curvature and roughness. The SMW also shows randomly curved regions locally and globally as shown in Figure 2b. The displacements estimated by the point cloud analyses can be over-or under-estimated by these local and global curvatures. The point cloud of the sheet pile shows the distribution of points that are flatter than the other two retaining structures (see Figure 3c). However, surfaces having different angles exist together in one sheet pile. If the angle of the surface and the direction in which the displacement occurs are not parallel to each other, a displacement error can be generated during the point cloud analysis [33]. Although the surface of the point cloud is flat, it is necessary to consider errors that can occur due to the angle of the surface.

### 3.2. Roughness Analysis for Each Retaining Structure

The displacement calculation of the point cloud is influenced by the roughness and curvature of the point cloud [34]. Therefore, in this paper, the roughness of the retaining structures was evaluated before the distance analysis of the point clouds obtained from each retaining. The roughness detection method by the kernel was applied to estimate the three-dimensional roughness in this paper. A kernel with a radius of ‘r’ that is set moves the point cloud and the distance between the kernel and the point can be calculated. The location of the kernel is determined where the average distance between points located inside the kernel and the kernel is minimized. The roughness is determined by the average value of the distance between the kernel and points, and the equation for calculating the roughness is shown in Equation (1) (CLOUDCOMPARE, [35]).

(1) $$R_{j}=\left(\left|\Delta Z_{1}\left|+\right|\Delta Z_{2}\left|\ldots \right|\Delta Z_{i}\right|\right)/n$$

where, *R* is roughness calculated for points within a kernel radius of *j*, Δ*Z* is the difference in elevation from a measurement point and the least-squares plane is calculated by the nearest neighboring measurement points and n is the number of measurement points within a kernel radius. 

**Figure 4:**
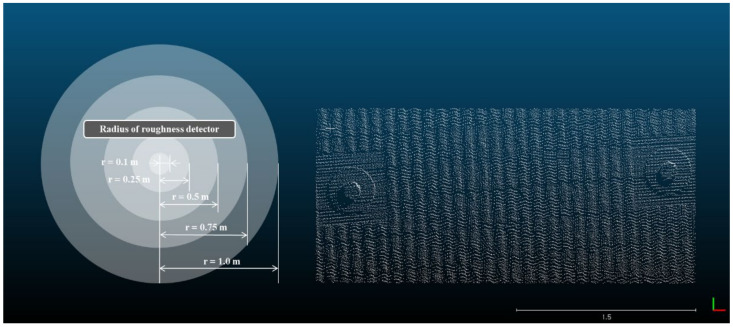
Estimation of roughness using kernel.

In this paper, the roughness was analyzed by increasing the radius of the kernel to 0.1 m, 0.25 m, 0.5 m, 0.75 m, and 1.0 m to evaluate not only the local roughness but also the global curvature of the retaining structures (see Figure 4). Small kernel size is suitable for detecting local roughness, and a large kernel size is suitable for detecting global curvature. As the radius of the kernel is smaller, the result is determined by the local roughness of the retaining structures within the radius of the kernel, and as the radius of the kernel increases, the result is influenced by the global shape change of the retaining structures. Therefore, the analysis according to the change of the kernel radius was performed to evaluate the effect of roughness and curvature on the distance analysis of retaining structures.

**Figure 5:**
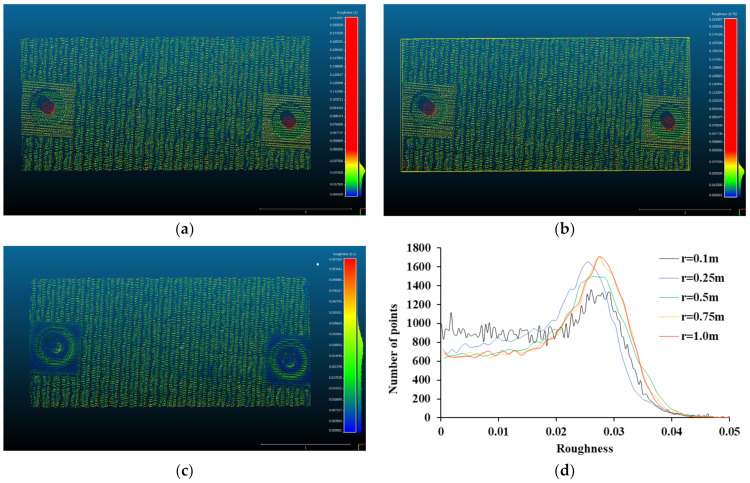
Roughness of concrete panel, (**a**) Kernel radius = 1.0 m, (**b**) Kernel radius = 0.5 m (**c**) Kernel radius = 0.1 m, (**d**) Histogram of roughness.

Figure 5 shows the result of roughness measurement using the kernel. Since different roughness can be evaluated according to the size of the kernel, the effect of the kernel size was considered in this result. When the radius of the kernel is 1.0 m, the estimated roughness is greatly influenced by the global curvature. Therefore, most of the roughness value was mainly reflected by the influence of the anchor shape different from the concrete panel shape. The zigzag shape and roughness of the concrete panel are not mainly expressed in the overall roughness scale (see Figure 5a). Most of the points expressing the change in the shape of the anchor are concentrated in the roughness of 0.18 m, and hence, in this paper, the effect of the anchor shape is filtered and results with roughness less than 0.05 m are analyzed. The influence of the anchor shape is dominant even when the radius of the kernel is 0.5 m (see Figure 5b). By filtering out the effect of the anchor shape, the roughness of the points is concentrated around 0.0273 m when the radius of the kernel is 1.0 m and 0.0263 m when the radius of the kernel is 0.5 m. Since the concrete panel has a zigzag shape, the roughness representing this curvature is evaluated at around 0.027 m. When the radius of the kernel is 0.1 m, the roughness at which points are most distributed is around 0.028 m. Therefore, the zigzag shape is reflected in the result at all kernel sizes. As shown in Figure 5c, a kernel radius of 0.1 m can eliminate the effect of shape by itself because the anchor face is not able to be covered by the kernel. It also better reflects the local roughness of the concrete panel as well as the zigzag shape compared to other results. Figure 5d shows the histogram of roughness at different kernel sizes. As the kernel size decreases, the number of points with roughness less than 0.02 m is increased. The local roughness can be more effectively detected by the smaller kernel, and the concrete panel shows a high value for both the zigzag shape and the local roughness. The roughness value is approached zero if t, the surface of the point cloud is smoother. However, the concrete panel has the highest number of points at about 0.027 m, with no significant change in the roughness value from 0 m to 0.02 m. Therefore, in the concrete panel, not only is the roughness value due to the local roughness is high, but also the roughness value due to the bending is high.

**Figure 6:**
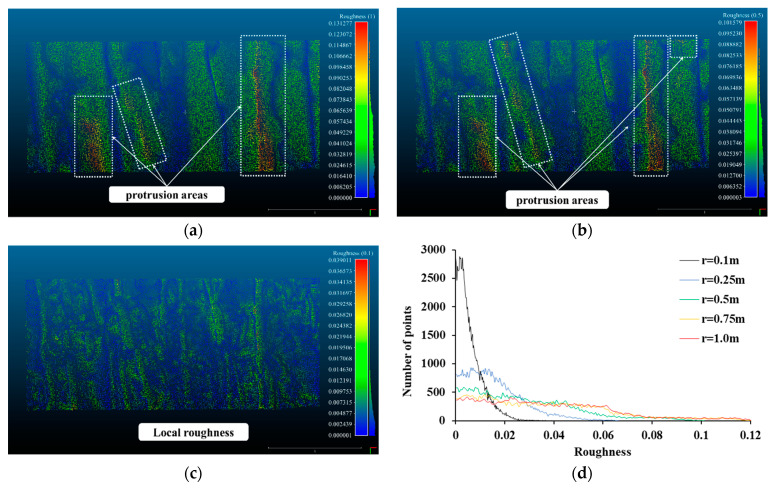
Roughness of soil mixing wall, (**a**) Kernel radius = 1.0 m, (**b**) Kernel radius = 0.5 m, (**c**) Kernel radius = 0.1 m, (**d**) Histogram of roughness.

Figure 6 shows the roughness analysis result of SMW by the kernel. When the kernel size is 1.0 m, the protrusion areas of the SMW show a high roughness value (0.0614 m), and the mean roughness value is 0.0378 m. The protrusion areas of the excavation surface in the SMW randomly formed by excavation are generally perceived as a 1.0 m kernel (see Figure 6a). However, local roughness is not detected in a kernel of 1.0 m. Even at a kernel of 0.5 m, the global curvature of the protrusion area is still revealed to be more detailed, but local roughness is not detected in detail (see Figure 6b) and the mean roughness value is 0.0277 m. However, at a kernel radius of 0.1 m, the roughness value due to global bending disappeared and the roughness values due to local roughness are expressed. The mean roughness value is 0.0049 m in a kernel of 0.1 m. Local roughness that was not revealed in the kernel results of 0.5 m and 1.0 m, is expressed on the entire surface of the SMW as shown in Figure 6c. Therefore, both global curvature and local roughness were measured in the SMW according to the size of the kernel. The detailed results of the roughness histogram of SMW according to each kernel size are shown in Figure 6d. When the kernel radius is 0.5 m to 1.0 m, the number of points is not decreased even when the roughness increases from 0 m to about 0.06 m. Since there are many points in the kernel size where global curvature can be found, the surface of the SWM has global curvature and needs to be considered in displacement analysis. As the radius of the kernel decreases from 0.25 m to 0.1 m, the points exist between the narrow roughness sections less than 0.02 m, which means that local roughness can be detected on the surface of SWM. Therefore, the surface of the SMW is formed with both local roughness and global curvature.

**Figure 7:**
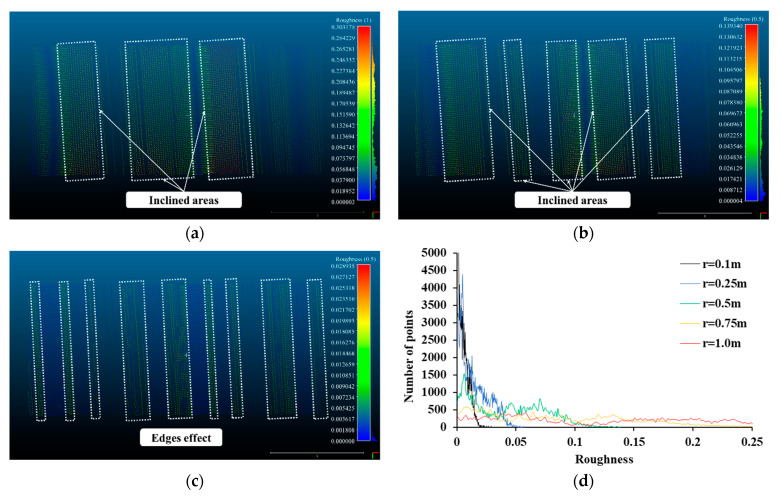
Roughness of sheet pile, (**a**) Kernel radius = 1.0 m, (**b**) Kernel radius = 0.5 m, (**c**) Kernel radius = 0.1 m, (**d**) Histogram of roughness.

The results of the kernel analysis of sheet piles are shown in Figure 7. The areas with large roughness values for a kernel radius of 1.0 m are clearly demarcated in Figure 7a. Since the sheet pile is divided into an inclined area and a flat area, the roughness value is greatly increased on the inclined side. Since the entire surface is covered by the kernel radius of 1.0 m, the inclined area is clearly revealed as roughness. The points of the inclined surface are distributed between 0.2 m and 0.3 m and the mean roughness value is 0.1203 m. The flat area has a low roughness value, and both areas are clearly distinguished by a kernel radius of 1.0 m. Both areas are clearly distinguished by a kernel radius of 0.5 m rather than the local roughness and the mean roughness value is 0.0430 m (see Figure 7b). However, when the kernel radius is reduced to 0.1 m, the roughness result is significantly different from the previous two cases because both areas are not entered within the kernel radius (see Figure 7c). Roughness values of most points are closed to 0 within each inclined and flat area, but the roughness value increases at an edge between the two areas. Therefore, the local roughness is smooth as close to 0, and the roughness value is increased by the edge effect. Figure 7d shows the histogram of roughness regarding the kernel radius, which shows the roughness trend of the sheet pile surface clearly. When the kernel radius is greater than 0.5 m, the points are distributed over a wide roughness section. The inclined surface and the flat surface are well distinguished. However, when the kernel size is 0.1 m, most points show a roughness value close to 0. This means that the kernel and the points are adjacent, indicating that the local roughness is smooth.

**Figure 8:**
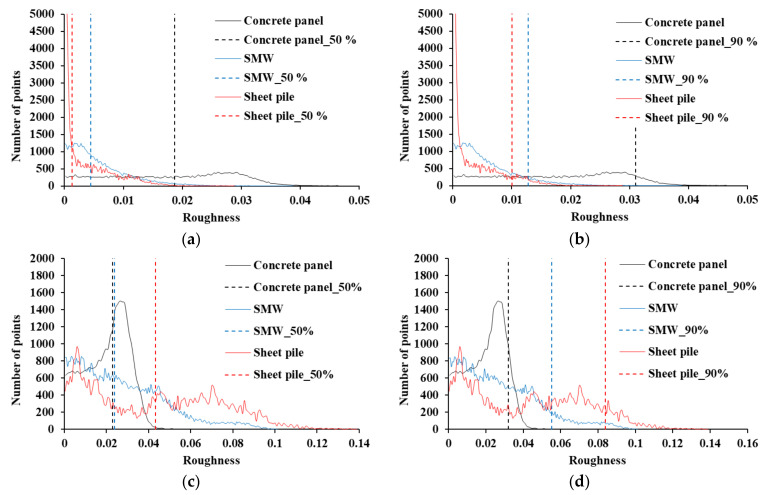
Local roughness and global curvature, (**a**) 50% of points (r = 0.1 m), (**b**) 90% of points (r = 0.1 m), (**c**) 50% of points (r = 0.5 m), (**d**) 90% of points (r = 0.5 m).

In order to compare the roughness behavior of the three retaining structures, the roughness histograms were compared as shown in Figure 8 when the kernel radii are 0.1 m and 0.5 m. In this analysis, the roughness characteristics of each retaining structure can be quantified. Figure 8a shows the roughness values corresponding to 50% of all points in the roughness histogram of the kernel radius of 0.1 m for each retaining structure. In the results of the kernel radius of 0.1 m, the local roughness was found to be 0.0187 m, 0.00442 m, and 0.00126 m for the concrete panel, SMW, and sheet pile, respectively. Therefore, the concrete panel had the highest local roughness, and it was found to be 4.2 times and 14.8 times rougher than the SMW and sheet piles, respectively. Figure 8b shows the roughness values corresponding to 90% of the points in the roughness histogram of the kernel radius of 0.1 m for each retaining structure. The roughness values corresponding to 90% of the points of the concrete panel, SMW, and sheet piles were 0.0301 m, 0.0100 m, and 0.0128 m, respectively. This result also shows that the local roughness is large in the order of sheet pile, SMW, and concrete panel. Therefore, in the point cloud analysis, the concrete panel needs to be considered by the local roughness. In the result of 0.5 m kernel radius, the roughness values corresponding to 50% of the points of the concrete panel, SMW, and sheet piles were calculated as 0.0230 m, 0.0239 m, and 0.0838 m, respectively (see Figure 8c). The roughness value of 50% is similar to the results of the concrete panel and SMW, but the roughness value of 90% clearly shows the features of the three retaining structures due to the global curvature. As shown in Figure 8d, the roughness values corresponding to 90% of the points of the concrete panel, SMW, and sheet piles were 0.0319 m, 0.0552 m, and 0.0838 m, respectively. The kernel radius of 0.5 m shows the largest value due to the shape change of the sheet. SMW is also affected by the mixture of concrete and ground that protrudes randomly, but the results of concrete panels are not reflected in global curvature as roughness compared to other retaining structures in a kernel radius scale of 0.5 m. Therefore, in the displacement analysis, the effect of the global bending of the sheet pile should be considered, and the influence of the randomly protruding area should also be considered for the SMW.

### 3.3. Azimuth Angle Analysis

**Figure 9:**
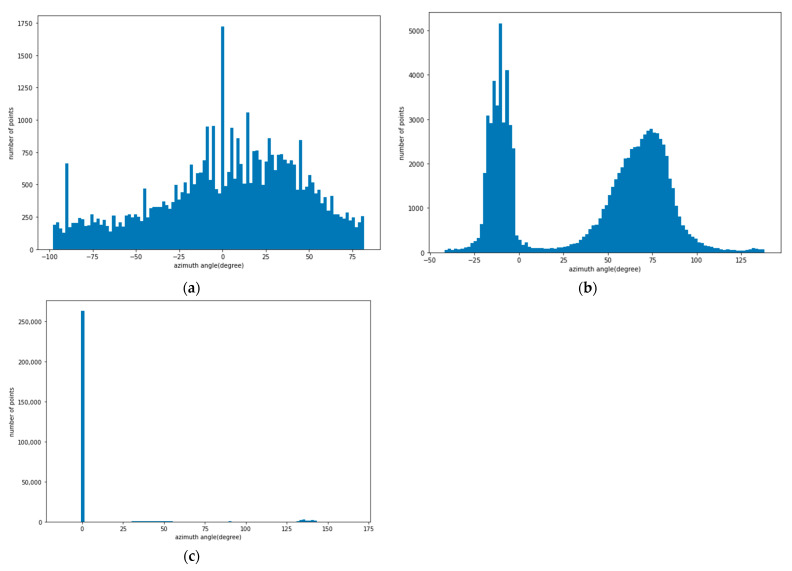
Azimuth angle distribution of points; (**a**) Concrete panel; (**b**) Soil mixing wall; (**c**) Sheet pile.

In order to analyze the curvature of the three retaining structures, the azimuth angles of each point were evaluated. An azimuth angle close to 0 means that it coincides with the directionality of the entire surface, and an increase or decrease of the azimuth angle means the curvature increases. Figure 9 shows the histogram of the azimuth distribution of each point in the point cloud of the three retaining structures. Since the entire surface of the concrete panel has a zigzag shape, the points are evenly distributed (see Figure 9a). In the SMW, the points are intensively distributed around −10 ° and 75 ° of azimuth angles (see Figure 9b). Since the surface of SMW is randomly curved, the histogram also shows a random distribution pattern. In the sheet pile, the points are concentrated around a 0° azimuth angle and the rest are distributed around 47° and 137° azimuth angles (see Figure 9c). Points distributed around 0° azimuth are points representing a flat surface, and the remaining points represent inclined surfaces. As shown in the azimuth analysis results, the sheet pile is affected by global curvature, the concrete panel is affected by local curvature, and SMW is affected by random curvature.

## 4. Displacement Error Evaluation

### 4.1. Displacement Error Calculation Method

**Figure 10:**
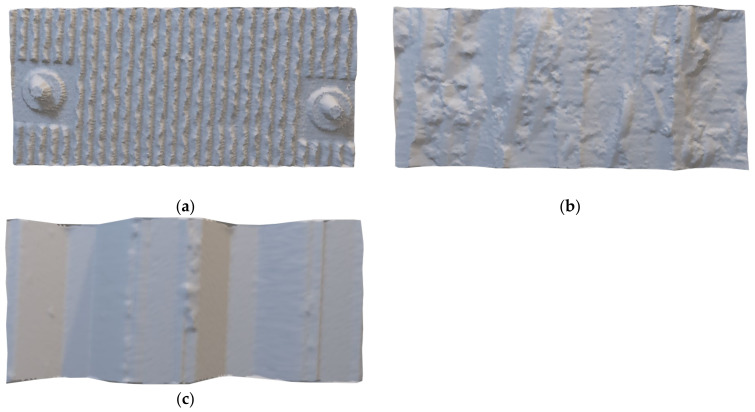
Meshes of each retaining structure, (**a**) Concrete panel; (**b**) Soil mixing wall; (**c**) Sheet pile.

In this paper, displacement was calculated using C2C, C2M, and M3C2 (Multiscale Model-to-Model Cloud Comparison) methods. In order to apply the C2M and M3C2 methods, the point cloud shown in Figure 3 needs to be converted into a three-dimensional mesh as shown in Figure 10. The points of the collected point cloud are connected to each other to create a triangular mesh. Figure 10a clearly shows the curvature and roughness of the mesh in the concrete panel. Figure 10b shows a randomly curved area with the mesh of SMW. Sheet pile is also clearly represented with flat and inclined areas (see Figure 10c).

**Figure 11:**
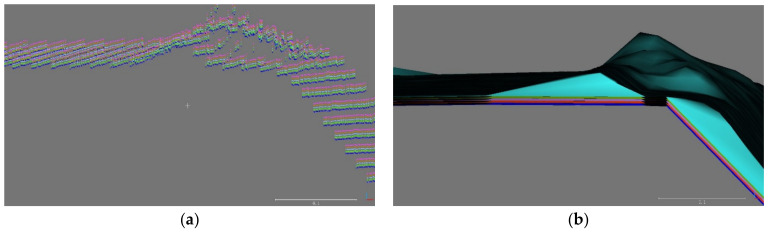
Example of artificial distance, (**a**) Shifted point clouds, (**b**) Shifted meshes.

In the point cloud displacement calculation, all three comparison methods are affected by the resolution of the point cloud. In order to consider the effect of the resolution, each point cloud as shown in Figure 3 was randomly divided into A and B groups by 50% of points. Group A is a point cloud that is a standard in displacement calculation, and group B is a point cloud that is for comparison in displacement calculation. For both groups, A and B, 100%, 80%, 60%, 40%, and 20% point clouds were randomly extracted. A point cloud was extracted from group A, and 19-point clouds were randomly extracted from group B. After all point clouds were placed parallel to the x-y axis, the 19-point clouds extracted from group B were artificially shifted to the *z*-axis except for one point cloud extracted from group A as a reference. The shifted distances are 0 mm, 2.5 mm, 5 mm, 7.5 mm, and 10 mm (see Figure 11a,b). The distance can be calculated by performing C2C, C2M, and M3C2 analysis between the reference point cloud and the remaining 19-point clouds. For each extracted percentage, 20 samples were compared, and the displacement error was calculated by comparing the artificial distance with the distance calculated by the C2C, C2M, and M3C2 methods. Each retaining structure may have various errors due to the various surface conditions. Therefore, the error calculated in this paper is the displacement calculation error that could occur in each retaining structure, rather than finding a direct correlation with the roughness or curvature of each retaining structure.

### 4.2. Estimations of Displacement and Displacement Error in Three Retaining Structures

**Figure 12:**
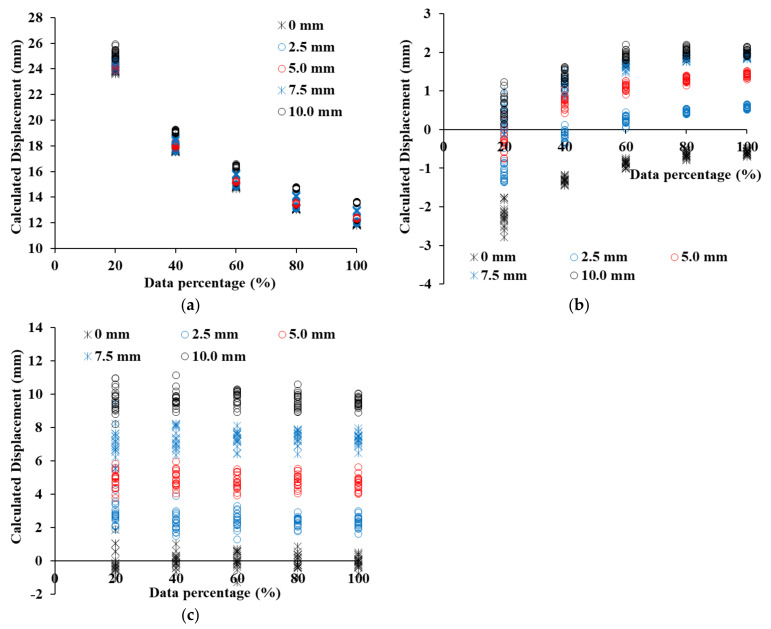
Displacement estimation in a concrete panel, (**a**) Displacement calculated by C2C, (**b**) Displacement calculated by C2M, (**c**) Displacement calculated by M3C2.

Figure 12 shows the displacement results calculated by C2C, C2M, and M3C2 when the point clouds of 19 concrete panels of group B are moved by 0 mm, 2.5 mm, 5.0 mm, 7.5 mm, and 10.0 mm. Based on the results analyzed in Figure 12, the average error was calculated as shown in Table 1. Despite not moving group B of the concrete panel, the displacement estimated by the C2C method increases from 11.90 mm to 23.92 mm from 100% point cloud to 20% point cloud (see Figure 12a). Since the C2C method calculates the distance between the closest points, it is significantly affected by the resolution compared to other methods [36]. Even if the displacement is increased, most of the displacements estimated by C2C obtain similar results. Although the C2M displacement increases, the displacement calculated by C2M gradually decreases and has a negative value. At a shifted displacement of 0 mm, an error is less than 1 mm, but an error of more than 8 mm is shown at a shift displacement of 10 mm. Even when the displacement is increased from 0 mm to 10 mm, the C2M displacement is not increased significantly from 0 mm (see Figure 12b). The reason is due to the zigzag shape of the concrete panel, which we will analyze in detail in Section 4.3. It will be analyzed in detail in Section 4.3. M3C2 displacement has an error of 0.1 mm or less when the shifted displacement is 0 mm, and has an error of 0.5 mm or less even when the displacement is increased. The M3C2 displacement reflects well the tendency of shifted displacements, of 0 mm, 2.5 mm, 5.0 mm, 7.5 mm, and 10.0 mm, as shown in Figure 12c. The 19 point clouds included in a group are intensively distributed with consistent values for each shift displacement.

**Figure 13:**
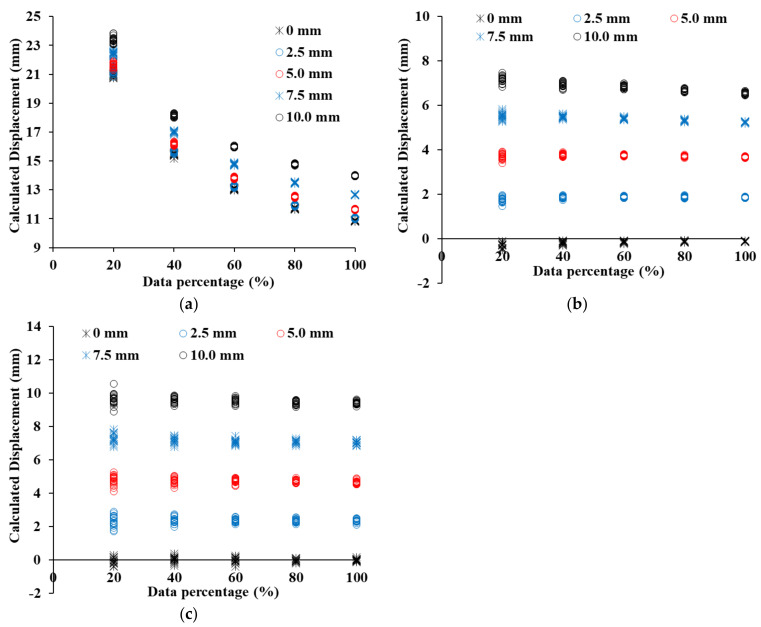
Displacement estimation in SMW, (**a**) Displacement calculated by C2C, (**b**) Displacement calculated by C2M, (**c**) Displacement calculated by M3C2.

Figure 13 shows the C2C, C2M, and M3C2 distance calculation results for the point cloud of SMW, and Table 2 shows the displacement error of each method. The C2C displacement calculation result has a constant C2C distance irrespective of the shifted displacement, just like the concrete panel (see Figure 13a). Therefore, as the shifted displacement increases, the error decreases. However, the C2M displacement shows a constant increasing pattern as the shifted displacement increases (see Figure 13b). At a shifted displacement of 0 mm, the error is −0.11 mm at 100% of the point cloud, and −0.33 mm at 20% of the point cloud. As the displacement increases, the error gradually increases, and at a shifted displacement of 10 mm, the error increases to −3.44 mm. The displacement is underestimated due to randomly protruding the shape in the SMW. A detailed analysis of the error generation according to the shape of the SMW will also be dealt with in Section 4.3. M3C2 was found to have the lowest error in SMW as well (see Figure 13c). For the displacement shifted by 0 mm, the maximum error was −0.06 mm in the point cloud of 20%, and the error occurred to less than −0.58 mm in the displacement of 10 mm shifted. The global curvature by protrusions exists on the surface in SMW and hence it is important to minimize errors in this region. M2C2 has a lower error than other methods because it calculates the displacement in the normal direction of the point cloud within the selected area at the protruding area.

**Figure 14:**
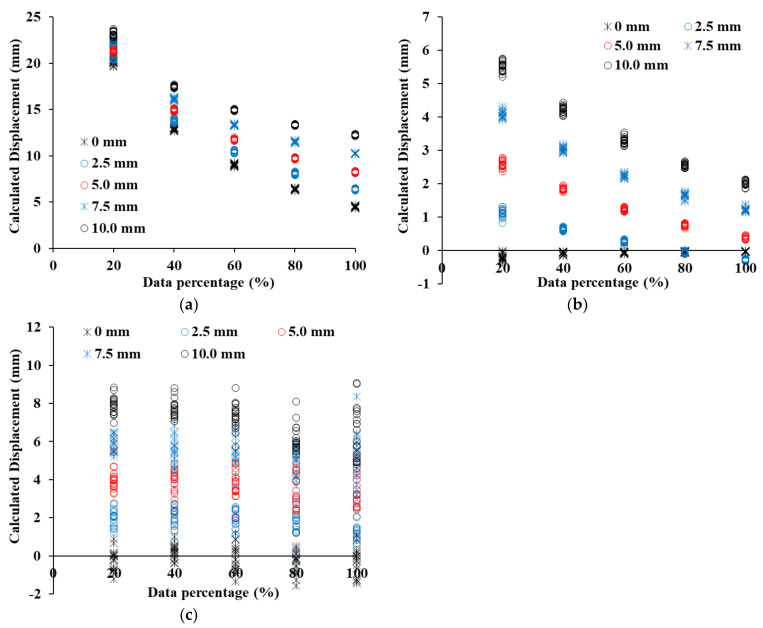
Displacement estimation in Sheet pile, (**a**) Displacement calculated by C2C, (**b**) Displacement calculated by C2M, (**c**) Displacement calculated by M3C2.

Figure 14 shows the displacement calculation results of C2C, C2M, and M3C2 in the sheet pile, and Table 3 shows the error of each method. Although the sheet pile has a flat surface, the C2C displacement shows a large difference from the shifted displacement. However, unlike the results of other retaining structures, the 100% point cloud has 4.48 mm at 0 mm shifted displacement and 2.24 mm at 10 mm shift displacement (see Figure 14a). As the shifted displacement increases, the C2C displacement increases at regular intervals. However, as the percentage of the point cloud decreases, the tendency to increase the displacement gradually decreases, indicating that the error gradually increases. A point cloud of 20% has an error of 20.15 mm at 0 mm shifted displacement and an error of 13.15 mm at 10 mm shifted displacement. For sheet piles, the error decreases rapidly as the resolution of the point cloud decreases. The C2M displacement has an error of less than 0.20 mm at 0 mm shifted displacement, but the error increases significantly as the shift displacement increases (see Figure 14b). In particular, the error of a 100% point cloud is larger than that of a 20% point cloud. As evaluated in the roughness analysis, the sheet pile had an inclined area, and hence the effect of global curvature is the greatest. The inclined area directly affects the error of C2M displacement, and a detailed analysis of this will be dealt with in Section 4.3. The error in the displacement of M3C2 in the sheet pile is larger than the error obtained in the displacement analysis of other retaining structures (see Figure 14c). The 0 mm shifted displacement result, not only has an error of 0.20 mm but also has a maximum error of −4.17 mm at 10 mm shifted displacement. The error varies randomly regardless of the percentage of the point cloud. The surface of the sheet pile consists of a flat area and an inclined area, and a sudden change in angle at the boundary between the two areas causes the M3C2 displacement error to increase. Therefore, in order to minimize the error due to the inclined area in the sheet pile, it is possible to minimize the error by extracting only the flat area and calculating the displacement.

### 4.3. Discussion of Analysis Methods

In Section 4.2, the displacement calculation and error of each retaining structure are identified, and in Section 4.3, the cause of the error is identified to suggest ways to improve the accuracy of C2C, C2M, and M3C2 displacement calculations. The C2C method showed the largest error in the displacement calculation of the three retaining structures. The error decreased as the shifted displacement increased, and the 100% point cloud showed the smallest error. Unusually, the error is smallest when the shifted displacement is 0 mm, but the C2C method shows the largest error in the 0 mm shifted displacement because it is greatly affected by the resolution of the point cloud. Table 4 shows the equivalent resolution calculated based on the number of points in the point cloud; 0 mm shifted displacement is likely due to an error of half the resolution because two different point clouds are entangled with each other. At different percentages of the point cloud, half of the equivalent resolution has a value similar to the error in the 0 mm shifted displacement. Therefore, it was confirmed that the influence of the resolution of the point cloud is dominant in C2C, and the C2C method as calculating the displacement of the three retaining structures can cause a large error due to the resolution.

**Figure 15:**
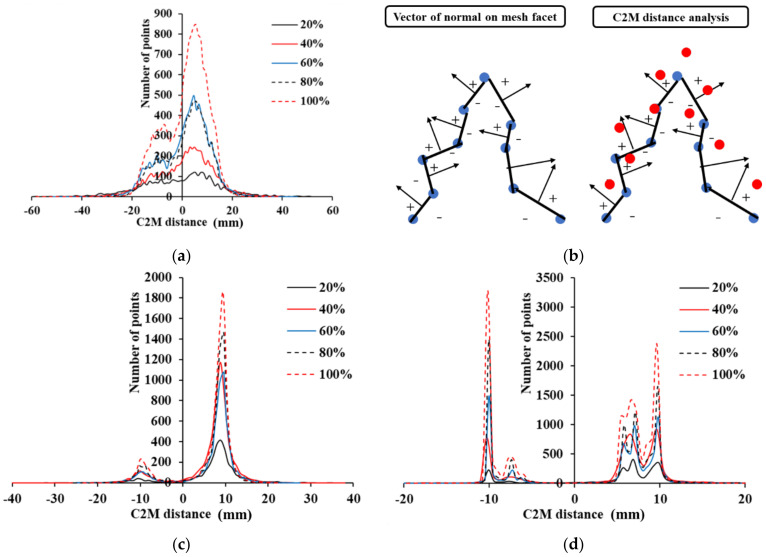
Causes of C2M Displacement Estimation Error, (**a**) C2M Histogram of concrete panel (10 mm shift),(**b**) Schematic diagram of direction of shifted distance in C2M, (**c**) C2M Histogram of SMW (10 mm shift) (**d**) C2M Histogram of sheet pile (10 mm shift).

In C2M analysis, a point cloud is converted into a mesh and calculates the C2M distance with another point cloud. The SMW of the three retaining structures showed the lowest error, and the other two retaining structures showed a small increase of the C2M displacement even when the shifted displacement increased in the results of Figure 12b, Figure 13b, and Figure 14b. In order to analyze this reason in-depth, the histogram of the C2M distance of each retaining structure was analyzed. Figure 15 shows the histogram results of the point cloud for each percentage at a shift distance of 10 mm. Figure 15a shows the C2M histogram results for a 10 mm shifted displacement in a concrete panel. The concrete panel has a rough surface and a zigzag shape. In Figure 15b, the blue point cloud is used to create the mesh, and the red point cloud represents the point cloud shifted in the z-direction for calculating distance. The normal vector of each facet in mesh, can be defined as shown in Figure 15b. The normal of a facet can be in two opposite directions which represent negative and positive distances. If the direction of the shifted displacement is perpendicular to the facet of the mesh, the positive displacement increases as the shifted displacement increases. However, on a curved surface, some point clouds can enter a region that is calculated as a negative value and can be calculated as a negative displacement. Therefore, when there is a curvature on the surface of the structure, it can have a negative value depending on the direction of displacement, which makes the mean C2M displacement underestimate. The C2M displacement in the histogram of the concrete panel shows peaks at 10 mm and −10 mm. Because the surface of the concrete panel is rough, the value around the peak is gradually decreased. Since the SMW has random curvature, most points represent C2M displacements of 10 mm, but some points in the randomly curved area show C2M displacements of −10 mm (see Figure 15c). Therefore, the C2M displacement is underestimated and errors become apparent. In the case of sheet piles, the C2M displacement is intensively distributed at 10 mm and −10 mm, and the C2M displacement of −10 mm is influenced by the direction of the mesh facet (see Figure 15d). The C2M displacement also shows small peaks around 7 mm and −7 mm, because the displacement is underestimated in the inclined area [37]. Therefore, in the sheet pile C2M analysis, the analysis should be performed after removing the inclined area. As shown in the results of C2M histograms in the three retaining structures, several points are distributed close to the shifted displacement, but there is an issue that some points show negative values due to the directionality of the mesh facet. Therefore, when analyzing the displacement of the retaining structure by C2M analysis, the influence of negative C2M displacement by the 3D axis must be removed.

**Figure 16:**
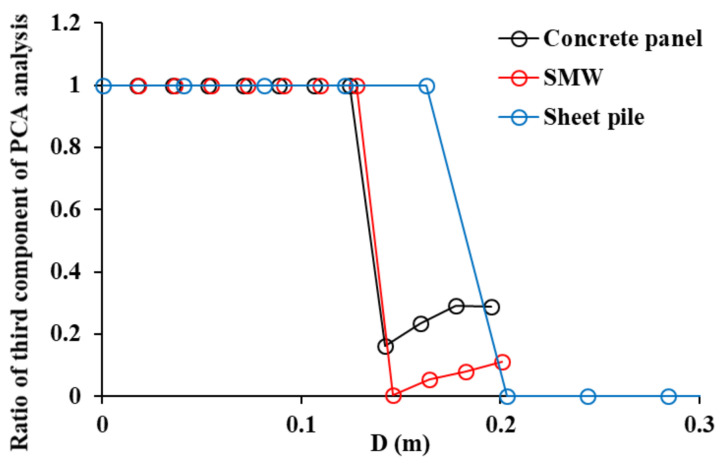
PCA analysis to define optimized D in M3C2.

M3C2 analysis had the highest accuracy in displacement calculations among the three analysis methods of retaining structures. Because M3C2 analysis is greatly affected by the diameter of the sphere, Principal Component Analysis (PCA) has to be performed before M3C2 displacement is calculated. A Principal Component Analysis was performed on the neighbors of point i within a sphere of radius D/2 and chose the Dopt scale at which the third component is the smallest. It is ensured that a minimum of 10 points is used to compute the normal at Dopt. Otherwise, the scale has to be selected at a larger size as soon as this requirement is met [32]. PCA analysis is applied to each sample. Examples show the variation of the third component with D for SMW at 100% data density; 1 is an artificial value that indicates the condition of 10 minimum points is not satisfied at the corresponding D value. The optimized D values for M3C2 analysis of concrete panel, SMW, and sheet pile were 0.142 m, 0.146 m, and 0.200 m, respectively (see Figure 16). Therefore, PCA analysis is essential in order to have high M3C2 displacement accuracy of retaining structures, especially with curvature and roughness. If the roughness, curvature, and density are similar in the entire point clouds, the PCA only needs to be performed once with some part of the point clouds. However, if these conditions change within a point cloud, the PCA must be performed again to estimate the D value.

## 5. Conclusions

The displacement of the retaining structure has to be strictly controlled during excavation and hence it is important to monitor the entire retaining structure. Although laser scanning can monitor the entire retaining structure in three dimensions, errors may occur depending on the condition of the surface and the displacement calculation method. Therefore, in this paper, the roughness and curvature analysis of the surface of the three retaining structures was conducted by the point clouds of concrete panels, SMWs, and sheet piles. The analysis error was also calculated when the C2C, C2M, and M3C2 methods were applied to the displacement calculation of each retaining structure. The detailed conclusion of this paper is as follows.

(1) in order to understand the roughness and curvature features of the point cloud in the three retaining structures, a roughness histogram analysis using the kernel was performed. In the result of the kernel radius of 0.1 m, the concrete panel had the highest local roughness, and it was found to be 4.2 times and 14.8 times rougher than the SMW and sheet piles, respectively. In the 0.5 m radius of the kernel where global curvature can be found, the sheet pile showed the largest value. Although SMW is also affected by the mixture of randomly protruding concrete and ground, the global curvature of the concrete panel is not reflected as roughness at a radius of 0.5 m compared to the other retaining structures. Therefore, in the displacement analysis, the effect of the global curvature of the sheet pile should be considered, and the influence of the randomly protruding area should also be considered for the SMW.
(2) the curvature of the retaining structure was analyzed with the histogram of azimuth angle, and in the concrete panel, the azimuth angles of the points are evenly distributed over the entire angle due to the zigzag-shaped surface. In the SMW, the azimuth angles of the points were intensively distributed around −10 ° and 75 °. In the sheet pile, the points are concentrated around 0° azimuth and the rest are distributed around 47° and 137° azimuth. In the azimuth analysis results, sheet piles are affected by global curvature, concrete panels are affected by local curvature, and SMW is affected by random curvature.
(3) the displacement and analysis errors of C2C, C2M, and M3C2 were calculated by shifting the 100%, 80%, 60%, 40%, and 20% point clouds of each retaining structure by 0 mm, 2.5 mm, 5.0 mm, 7.5 mm, 10.0 mm. The displacement calculated by C2C showed the largest error in all retaining structures, and it is confirmed that the error was determined by the resolution of the point cloud.
(4) a curved area existed in all three retaining structures, and the C2M displacement was underestimated because the negative C2M displacement was calculated according to the direction of the point in the curved section. Therefore, when analyzing the displacement of the retaining structure by C2M analysis, the influence of negative C2M displacement has to be removed.
(5) M3C2 analysis had the highest accuracy in displacement calculation among the three analysis methods for retaining structures. PCA analysis is essential in order to get high M3C2 displacement accuracy of retaining structures with curvature and roughness. The optimized D for the M3C2 analysis of concrete panel, SMW, and sheet piles introduced in this paper were 0.142 m, 0.146 m, and 0.200 m, respectively.

## Figures and Tables

**Table 1:** Displacement errors of C2C, C2M, and M3C2 in a concrete panel.

Displacement (mm)	Errors of C2C (mm)				
	20%	40%	60%	80%	100%
0	23.92	17.66	14.84	13.09	11.90
2.5	21.60	15.26	12.44	10.70	9.53
5	19.47	13.11	10.29	8.55	7.38
7.5	17.14	11.02	8.29	6.59	5.43
10	15.20	9.12	6.39	4.72	3.59
**Displacement (mm)**	**Errors of C2M (mm)**				
	**20%**	**40%**	**60%**	**80%**	**100%**
0	−2.20	−1.32	−0.89	−0.66	−0.61
2.5	−3.62	−2.67	−2.24	−2.03	−1.92
5	−5.27	−4.26	−3.88	−3.70	−3.58
7.5	−7.19	−6.29	−5.85	−5.65	−5.59
10	−9.39	−8.61	−8.08	−7.97	−7.99
**Displacement (mm)**	**Errors of M3C2 (mm)**				
	**20%**	**40%**	**60%**	**80%**	**100%**
0	−0.03	0.04	0.08	−0.03	−0.04
2.5	0.23	−0.12	−0.05	−0.14	−0.12
5	−0.11	−0.08	−0.19	−0.16	−0.34
7.5	−0.42	−0.15	−0.15	−0.11	−0.19
10	−0.31	−0.30	−0.25	−0.38	−0.49

**Table 2:** Displacement errors of C2C, C2M, and M3C2 in SMW.

Displacement (mm)	Errors of C2C (mm)				
	20%	40%	60%	80%	100%
0	21.05	15.45	13.07	11.72	10.86
2.5	18.72	13.13	10.75	9.42	8.52
5	16.66	11.18	8.84	7.51	6.64
7.5	14.96	9.50	7.28	5.99	5.18
10	13.38	8.15	5.99	4.77	3.98
**Displacement (mm)**	**Errors of C2M (mm)**				
	**20%**	**40%**	**60%**	**80%**	**100%**
0	−0.33	−0.19	−0.14	−0.12	−0.11
2.5	−0.74	−0.63	−0.62	−0.61	−0.64
5	−1.30	−1.23	−1.25	−1.29	−1.32
7.5	−1.98	−2.01	−2.09	−2.18	−2.25
10	−2.83	−3.07	−3.16	−3.32	−3.44
**Displacement (mm)**	**Errors of M3C2 (mm)**				
	**20%**	**40%**	**60%**	**80%**	**100%**
0	−0.06	0.01	0.00	−0.01	−0.01
2.5	−0.15	−0.12	−0.12	−0.15	−0.14
5	−0.23	−0.24	−0.26	−0.27	−0.33
7.5	−0.20	−0.33	−0.42	−0.40	−0.46
10	−0.34	−0.42	−0.46	−0.56	−0.58

**Table 3:** Displacement errors of C2C, C2M, and M3C2 in sheet pile.

Displacement (mm)	Errors of C2C (mm)				
	20%	40%	60%	80%	100%
0	20.15	12.86	9.01	6.39	4.48
2.5	18.08	11.30	7.94	5.58	3.86
5	16.43	9.95	6.78	4.75	3.26
7.5	14.88	8.63	5.82	4.00	2.76
10	13.15	7.49	4.93	3.33	2.24
**Displacement (mm)**	**Errors of C2M (mm)**				
	**20%**	**40%**	**60%**	**80%**	**100%**
0	−0.20	−0.08	−0.06	−0.04	−0.03
2.5	−1.36	−1.85	−2.23	−2.56	−2.79
5	−2.41	−3.15	−3.78	−4.24	−4.61
7.5	−3.42	−4.46	−5.25	−5.85	−6.27
10	−4.48	−5.78	−6.72	−7.45	−7.97
**Displacement (mm)**	**Errors of M3C2 (mm)**				
	**20%**	**40%**	**60%**	**80%**	**100%**
0	−0.19	0.20	−0.10	−0.31	−0.17
2.5	−0.50	−0.43	−0.36	−0.72	−0.84
5	−1.00	−1.10	−1.26	−1.53	−1.52
7.5	−1.62	−1.95	−1.91	−2.49	−2.13
10	−2.10	−2.30	−2.65	−4.17	−3.39

**Table 4:** Resolution of extracted point clouds.

Percentage	100%	80%	60%	40%	20%
number of points	15169	12135	9101	6068	3034
Area (m^2^)	4.63	4.63	4.63	4.63	4.63
density (points/m^2^)	3278	2622	1967	1311	656
Equivalent resolution (mm)	17.47	19.53	22.55	27.62	39.06

## Data Availability

Some or all data, models, or code generated or used during the study are available from the corresponding author by request. (Point cloud data).
